# Adventures in public data

**DOI:** 10.1186/1758-2946-3-34

**Published:** 2011-10-14

**Authors:** Dan W Zaharevitz

**Affiliations:** 1 National Cancer Institute/NIH, Bethesda, Maryland, USA

## Abstract

This article contains the slides and transcript of a talk given by Dan Zaharevitz at the "Visions of a Semantic Molecular Future" symposium held at the University of Cambridge Department of Chemistry on 2011-01-19. A recording of the talk is available on the University Computing Service's Streaming Media Service archive at http://sms.cam.ac.uk/media/1095515 (unfortunately the first part of the recording was corrupted, so the talk appears to begin at slide 6, 'At a critical time'). We believe that Dan's message comes over extremely well in the textual transcript and that it would be poorer for serious editing. In addition we have added some explanations and references of some of the concepts in the slides and text. (Charlotte Bolton; Peter Murray-Rust, University of Cambridge)

**Editorial preface:**

The following paper is part of a series of publications which arose from a Symposium held at the Unilever Centre for Molecular Informatics at the University of Cambridge to celebrate the lifetime achievements of Peter Murray-Rust. One of the motives of Peter's work was and is a better transport and preservation of data and information in scientific publications. In both respects the following publication is relevant: it is about public data and their representation, and the publication represents a non-standard experiment of transporting the content of the scientific presentation. As you will see, it consists of the original slides used by Dan Zaharevitz in his talk "Adventures in Public Data" at the Unilever Centre together with a diligent transcript of his speech. The transcribers have gone through great effort to preserve the original spirit of the talk by preserving colloquial language as it is used at such occasions. For reasons known to us, the original speaker was unable to submit the manuscript in a more conventional form. We, the Editors, have discussed in depth whether such a format is suitable for a scientific journal. We have eventually decided to publish this "as is". We did this mostly because it was Peter's wish that this talk was published in this form and because we agreed with his notion that this format transmits the message just as well as a formal article as defined by our instructions for authors. We, the Editors, wish to make clear however that this is an exception that we made because we would like to preserve the temporal unity and message of this set of publications. Insisting on a formal publication would have meant losing this historical account as part of the thematic series of papers or disrupting the series. We hope that this will find the consent of our readership.

## Introduction

**Figure 1:**
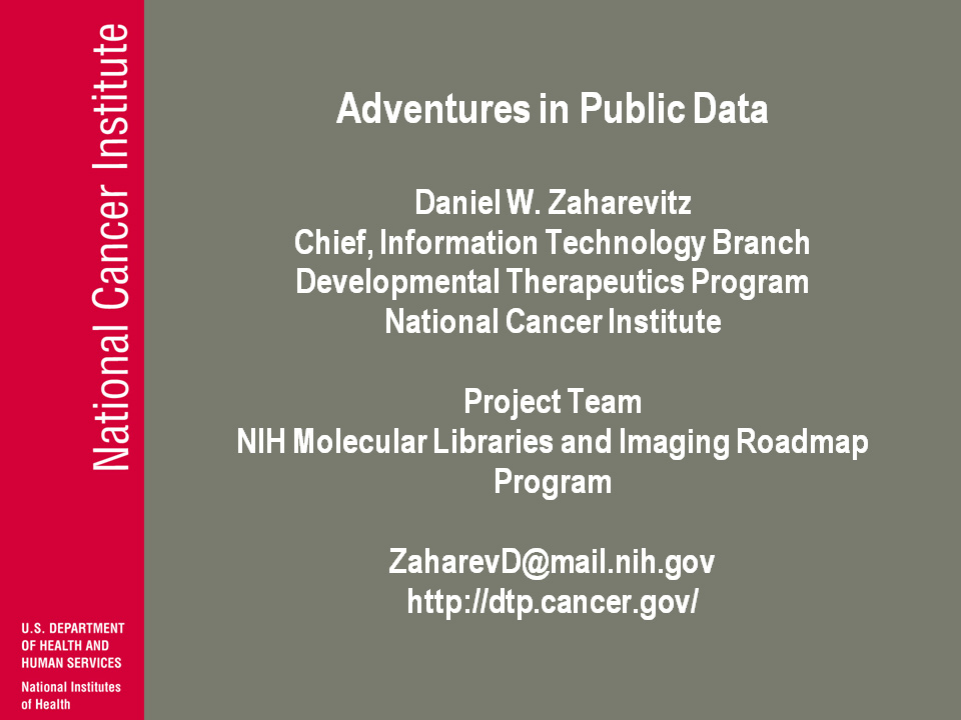
**Introduction**.

(Figure 1) This article contains the slides and transcript of a talk given by Dan Zaharevitz at the "Visions of a Semantic Molecular Future" symposium held at the University of Cambridge Department of Chemistry on 2011-01-19. A recording of the talk is available on the University Computing Service's Streaming Media Service archive at http://sms.cam.ac.uk/media/1095515 (Endnote 1). We believe that Dan's message comes over extremely well in the textual transcript and that it would be poorer for serious editing. In addition we have added some explanations and references of some of the concepts in the slides and text. (Charlotte Bolton; Peter Murray-Rust, University of Cambridge)

## Discussion

**Figure 2:**
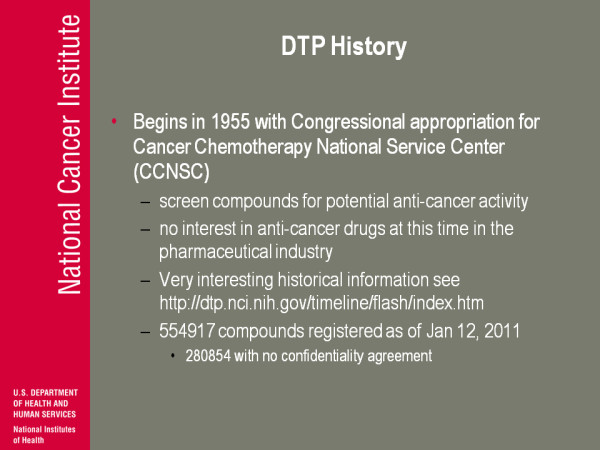
**DTP History**.

**Figure 3:**
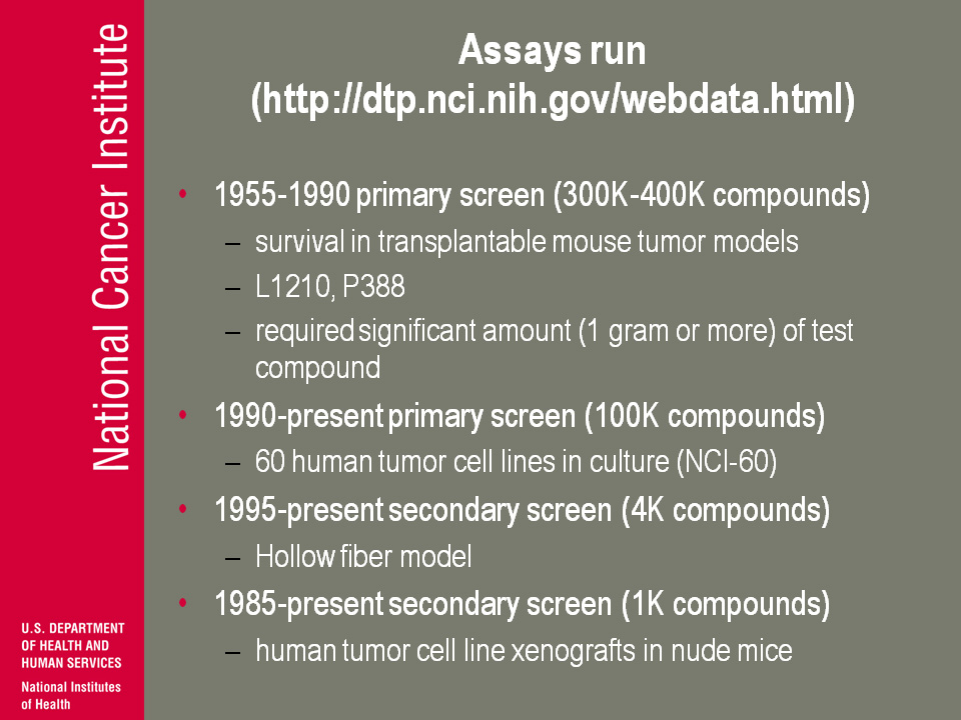
**Assays run**.

**Figure 4:**
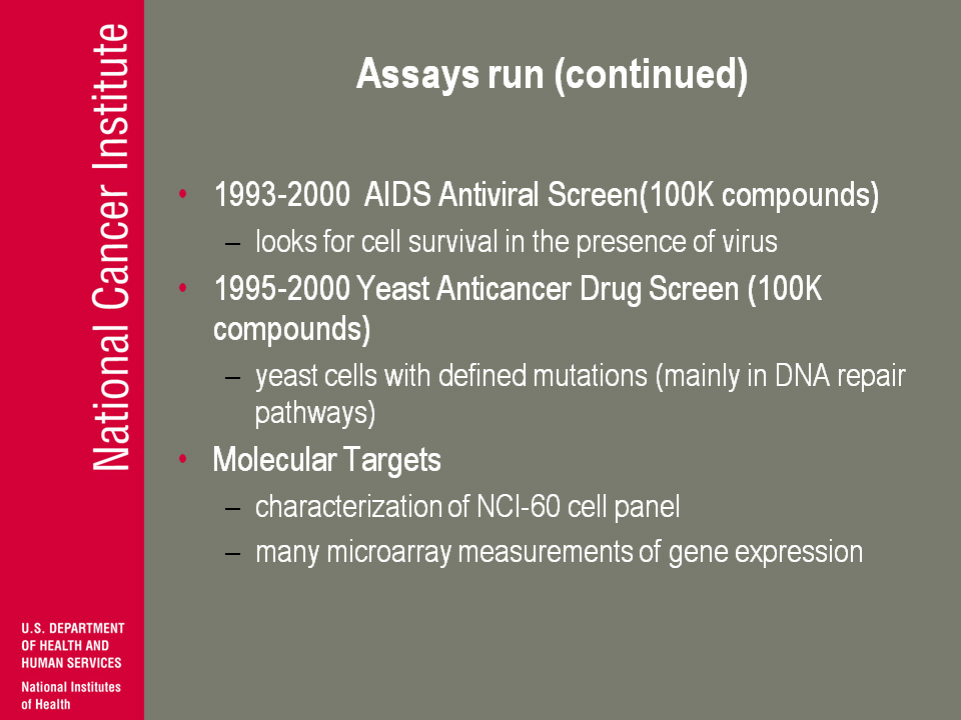
**Assays run (continued)**.

(Figure 2) The history of the DTP (Developmental Therapeutics Program) starts in 1955 with a US Congress specific appropriation to create a national chemotherapy service centre (Endnote 2). The rationale for this was that at the time there was no interest in attempting to develop anti-cancer drugs within the pharma industry. It was thought not possible to alter the course of the disease. Compounds were screened for anti-cancer activity. The primary screen was transplantable mouse models. Over the course of >50 years that the NCI has been acquiring compounds (Endnote 3), we have registered more than 550,000 compounds. Roughly half of these (280,000) were acquired without confidentiality agreement so that data can be publicly made available.

(Figures 3 and 4) What are the sources of data? This is all on the DTP webpage. For the majority of the history, the primary screens were L1210 and P388 mouse leukemias. Some 300,000-400,000 compounds were run through these models. The screens required significant amounts of the compound. We looked at preliminary toxicology, which involved lots of animals and lots of doses, so we needed them to get lots of compound-a gram or two. This has present day implications-if a compound was dropped early (e.g. not enough activity, too much toxicity), a large amount of the compound was left in our inventory. And this material is now publicly available.

In the 1980s it was publicly recognised that the mouse models were not general enough to pick up solid tumor agents that people were interested in. So we developed the human tumor cell-line inhibition screen, known as NCI-60. We've run roughly 100,000 compounds in last 20 years, and this screen is still active. There were a number of secondary screens dating from the mid90s to the present: hollow fibre model, where tumor cells are implanted in a semi-permeable fibre which is implanted in the mouse. Multiple fibres can be implanted in one mouse so it's possible to test multiple cell-lines per mouse. This gives us a hint of in vivo activity in an efficient and cost-effective assay. We also use human tumor xenografts in nude mouse: 1500-2000 screens in the last few years.

Because of the NCI infrastructure for acquiring and testing interesting compounds, with the sources of compounds and the data, and having the infrastructure already set up to test large scale compounds and assays when the AIDS epidemic hit, the screening for anti-HIV compounds ended up in DTP. In roughly 10 years 1990-2000, DTP assayed roughly 100,000 compounds in AIDS antiviral screens, looking for survival of cells in the presence of the virus.

There was also an attempt to create a yeast anti-cancer screen. This took yeast with known mutations, generally in the DNA repair pathway, and treated them with drugs, looking for toxicity for defined mutations. Specificity for a particular mutation gives mechanistic information.

**Figure 5:**
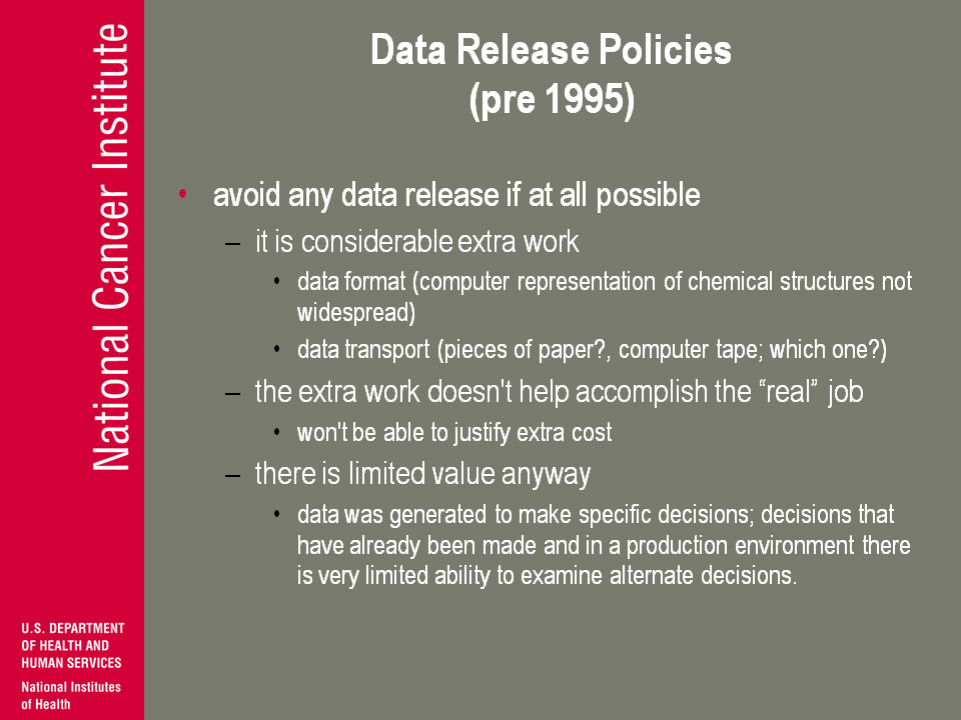
**Data Release Policies (pre 1995)**.

Lastly, with the NCI-60 cell-lines, there has been an effort to characterise all the cells in these panels in wide variety of ways. This effort is ongoing. We now have 8-10 separate measures using microarrays of gene expression, so there is lots of this available for NCI-60. It's very useful to correlate with growth inhibition patterns.

(Figure 5) So all of this created a large amount of data available to the public. Before 1995 the policy was to avoid data release if possible. The thinking behind this was several-fold. It created a lot of extra work. What data format do you use? The representation was not well settled, so it's problematic, even if you had a reasonable electronic representation, how was best to transport it to others? There are physical (tapes) and format considerations. It's very difficult to conceive of a way for widespread distribution of data without an awful lot of hands-on work. And the extra work doesn't help you to accomplish your 'real job'. The extra work doesn't get compounds into the clinic, or give you further information about how to do your job. It just sucks up time and resources.

**Figure 6:**
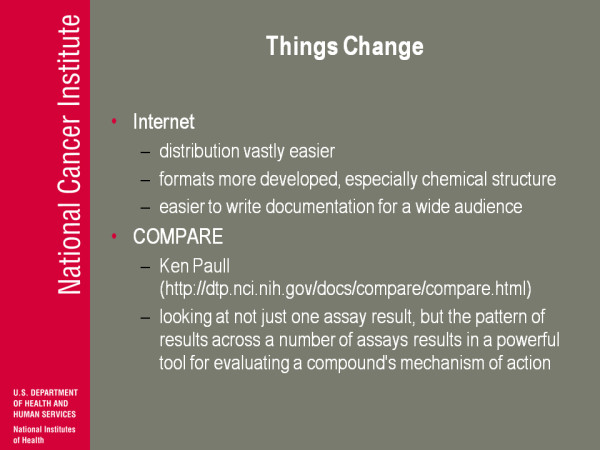
**Things Change**.

And it's of limited value anyway. The data was generated to make specific decisions, which are already made. It's a production environment, you can't easily examine alternate decisions. People from outside might ask-why didn't you do this? That's not wrong but in the production environment you have to make decisions and move on. The next couple of thousand compounds are on the way, you must move forward. Why look at data just to rehash a decision that couldn't be re-examined anyway?

(Figure 6) In the mid 1990s there were dramatic changes. These were driven by the development of the internet-now distribution is not an issue. You can quite easily distribute data to hundreds of thousands of people all over world with virtually no effort. The formats for chemical structure were more developed, more useful, embedded in software. It was easier to give people documentation. HTML made it much easier to give people a way to collect data with clear and accessible documentation for that data: what it is, how to use it etc. You could spend less time on the phone having to explain all this!

**Figure 7:**
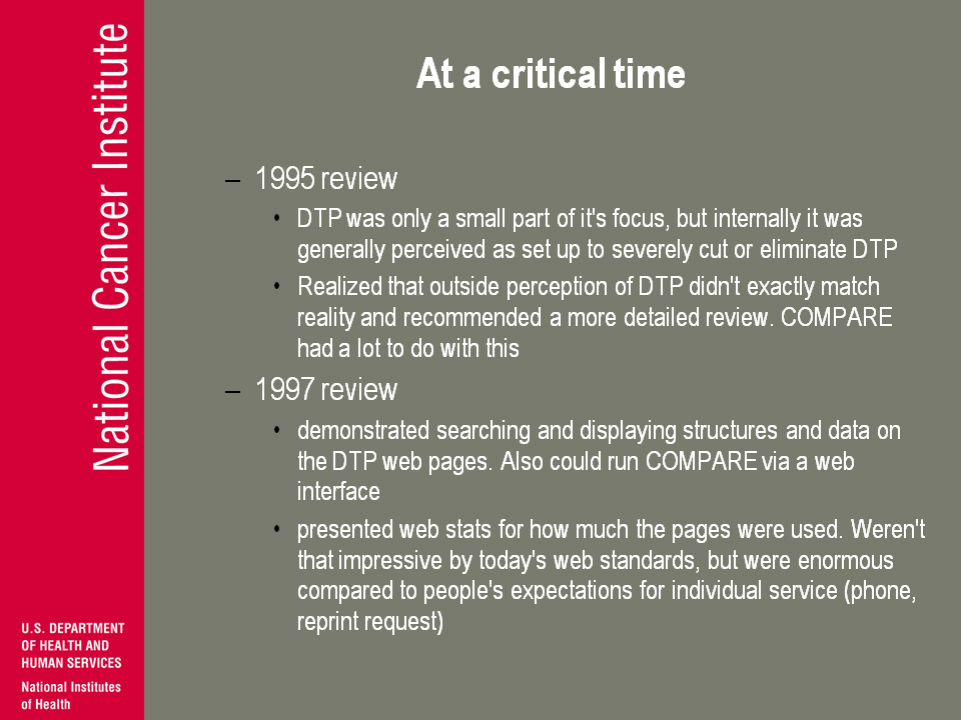
**At a critical time**.

Ken Paull (Endnote 4), former chief of the IT branch, developed COMPARE, which was looking at the NCI-60 cell-line data, not as individual assay results, is one cell-line sensitive another not, but at the overall pattern of activity. If the correlation was high between two compounds, it's likely to mean that the compounds shared the mechanism of action. It was a powerful tool to take a gross empirical assay to give a biochemical idea of what's going on. Using assay results as a pattern, to give an overall finger print of activity is a very powerful tool compared to looking at these things one at a time.

(Figure 7) And so the very detailed review came about. It was a year-long review-forty people-it was massive. And for the purposes here to talk about it I demonstrated searching and displaying structures and data on the DTP web pages and showed that you can also run COMPARE via your web interface. One of the big guns chased me out of the room where the presentation was given, was totally excited, he could see that you could sit in your living room or you could sit in your office and you could explore all kinds of ideas by just logging on to a web page, and in 1997 that wasn't exactly the most widespread notion in the world.

**Figure 8:**
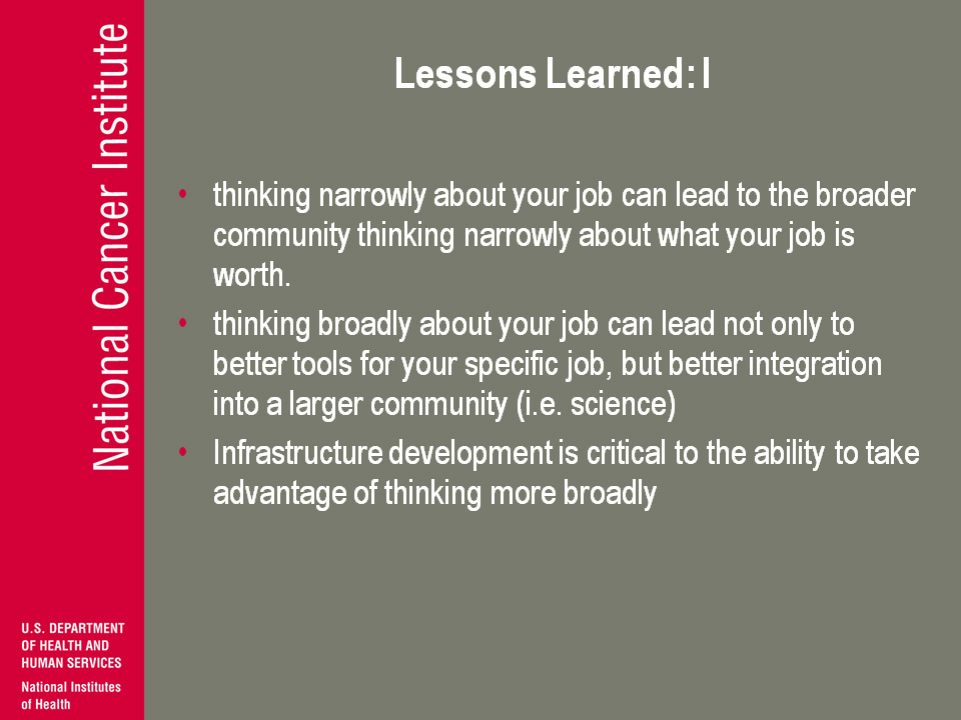
**Lessons Learned: I**.

**Figure 9:**
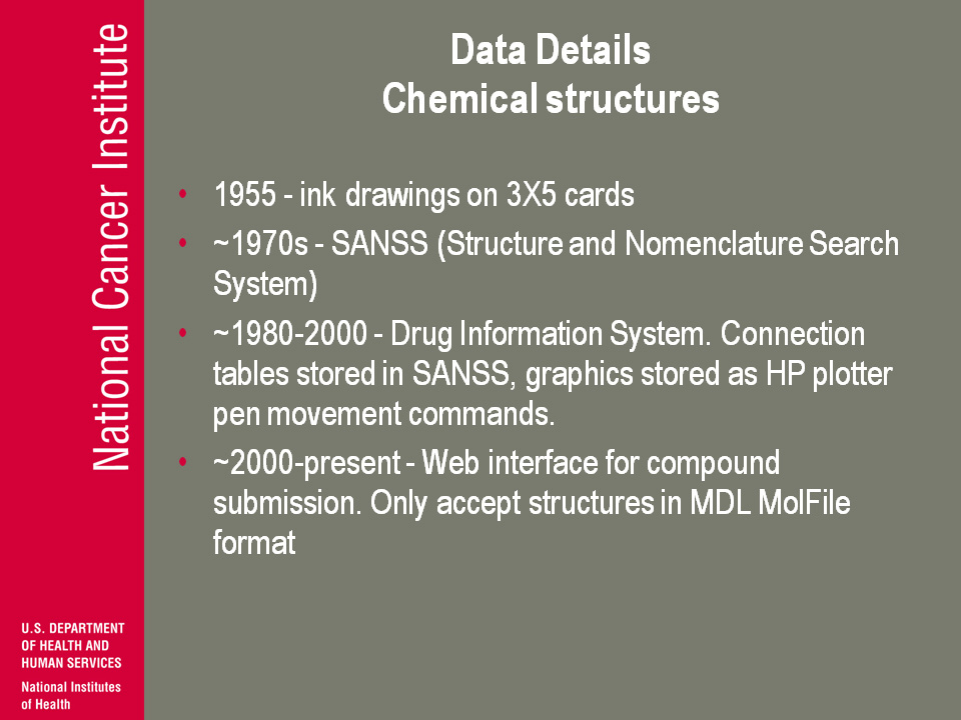
**Data Details Chemical structures**.

I also presented web stats for how many pages were accessed. The number of hits weren't that impressive by today's standards but again you're talking with people that were used to thinking of contacts as phone, reprint requests, fax requests, something like that and it was clear that your ability to respond to requests for outside information via a website was just enormous compared to the things you could think about in the 1980s. I also point out my boss at the time made the specific challenge to some of the people, the reviewers in the room, talking about the worth of the developmental therapeutics programme and asked them, you know, big drug company guys 'Can you point me to your web page where I can download your data?', and so it drives home the point that there was a difference.

(Figure 8) Lessons learned. I'm gonna call these lessons learned. You might say exaggerated extrapolations from limited knowledge but this is the thing that I take home from it. So if you think narrowly about your job, don't be surprised when the broader community thinks narrowly about what your job is worth. On the other hand if you think broadly about what your job is, it can lead to not only better tools for your specific job, you can be better at what you think you need to do but you also end up having better integration into the larger community. And a point that will come back to you time and time again is, infrastructure development is critical to enable the ability to take advantage of this broad thinking. All the good intentions in the world in the mid nineties would not have enabled us to do some of these things without the infrastructure of the internet.

(Figure 9) So I go now into some details of the chemical structures we collected (Endnote 5). You can download an SDF file from us and say great I'll take my structures and go on. Here is all the stuff I have to deal with to get it to an SDF file. So in 1955 collecting chemical structures meant sort of ink drawings on 3 × 5 cards; that's the beginnings of our compound collection. In the 1970s there was this SANSS (Structure and nomenclature search system) which essentially was a connection table format. It gave you the atoms and which atoms were connected and what the bond order was but it had no coordinate information, no display information. This I think was partly due to CAS. There was also the EPA NIH chemical information system that was coming about here. For about twenty years starting about 1980 we had what we called the drug information system. The connection tables were stored in CAS but you also had a picture. The picture was stored in the database as HP plotter pen movement commands, so you can get a picture, you can get a connection table but you couldn't put them together at least in any useful way.

**Figure 10:**
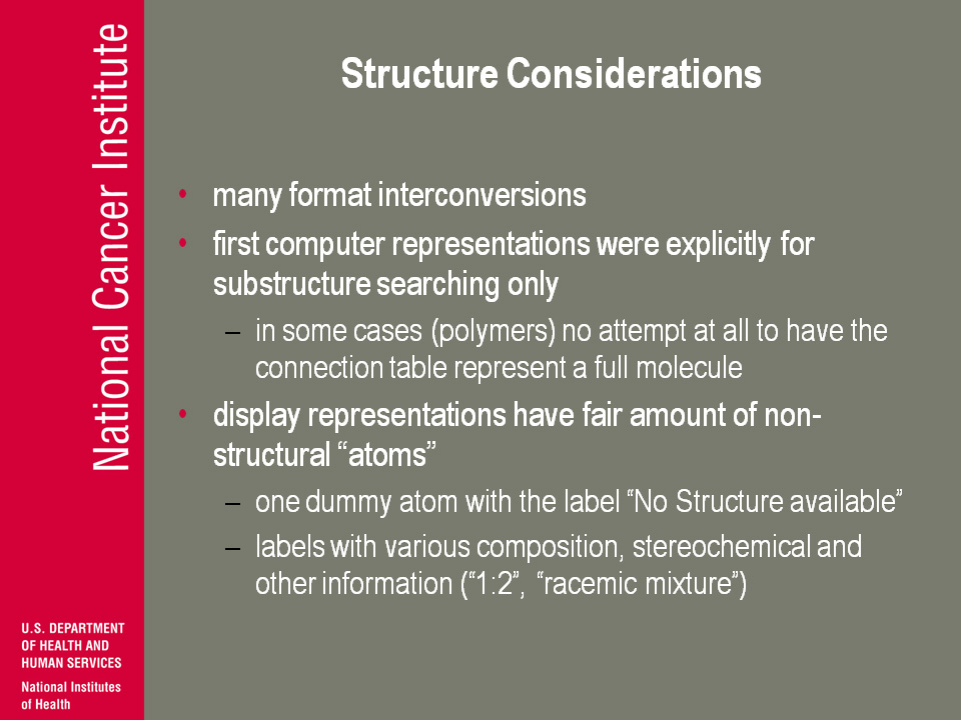
**Structure Considerations**.

From about 2000 to present we went to a fully integrated relational database, did all those conversions and right now we have an online submission where we only accept the structures at the moment in an MDL molfile format. At least from the beginning we have a computer representation that comes in. I should point out the entire time up to the institution of this online request system, the procedure for asking us to test a compound was the supplier would send in a picture, would send in a piece of paper, a graphic so we did not have an electronic interaction between the requester and our systems; it was all us doing transfers from some kind of picture.

(Figure 10) Considerations on what to do; how to get this into something we can make public. There were many, many format inter conversions throughout the fifty years this was going on. One thing to note, and I think it's again important, when you see it's easy to say here's a chemical structure, all chemical structures are alike, they all came from somewhere. If you don't understand where they came from you're not necessarily gonna understand what the strengths and weaknesses of various sets are. The first computer representation of the SANSS was explicitly for sub-structure searching. In some cases, for example polymers, there was no attempt to have the connection table represent a full molecule. The idea was you don't need to all that information if you're not gonna model; it was not for modelling, it was not for computing properties, it was for doing sub-structure searching. If you take a polymer, if you had say a dimer: most of the kind of substructure elements that you might search for are probably gonna be represented in the dimer. You can argue trimer or what not, but you don't need the whole thing because you're not gonna have a sub-structure that says search for a linear chain of 200 atoms or something like that. Most sub-structure searches are more limited so you don't need to bother to put the whole molecule in. So what you end up doing now is having perfectly wonderful SANSS files, that look perfectly complete, that in fact never ever had any intention of representing what that molecule was or what that substance was in a vial.

**Figure 11:**
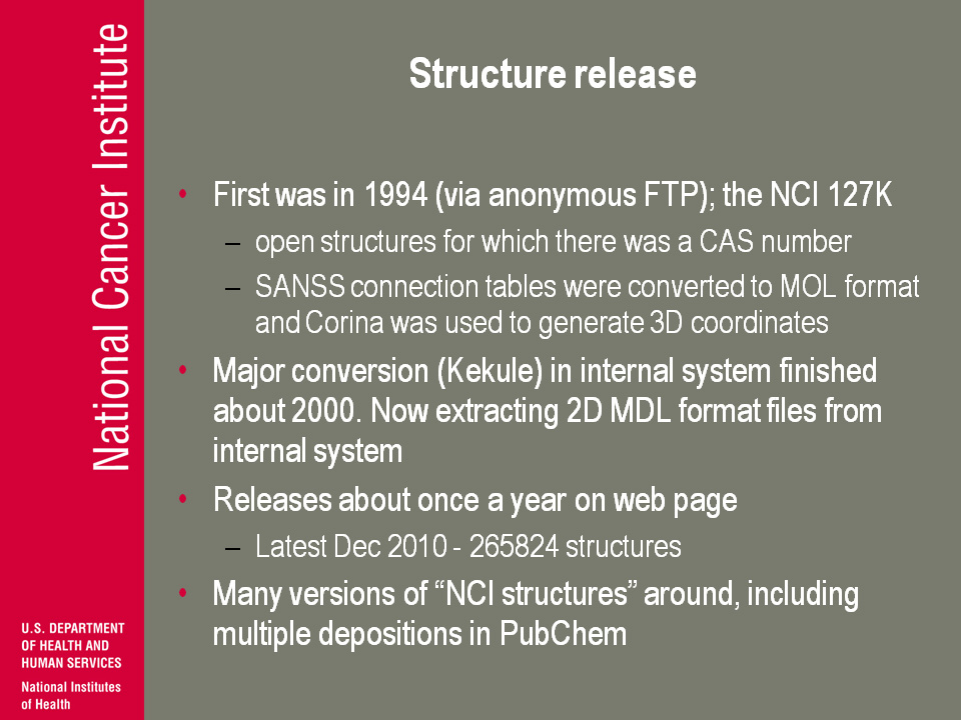
**Structure release**.

The display representations also had a fair amount of what you'd call non-structural features and the one that drives me up the wall today is a structure that comes out as a perfectly legal molfile which has one dummy atom with a label "no structure available". You know, I mean, enough said about that, it still drives me up the wall... But you also have labels so there is a dummy atom that has a label that actually has something that you might want to capture. So, composition of the two parts of the substance: label it as a racemic mixture, label it as something else so maybe you don't wanna completely just delete it,' but at the same time it's a pollution of the structure with other information in a format that's hard to disentangle.

(Figure 11) Structure release. The first one we put on the NIH page, not a page, just for anonymous FTP. I think it's generally called the NCI 127 k. They were open structures for which there was a CAS number. We figured the other thing to realise is that a lot of people say 'can you give us the chemical names of all these structures?' The vast majority of these structures were not published on, or at least we don't know that they were published on: no one ever bothered to name it, there's certainly not a trivial name. And so for a lot of the structures the only identifier we had was the NSC number and of course back in 1994 nobody knew what an NSC number was except for a handful of people interacting with NCI. We didn't think that was very useful so we sub selected a set where we also had the CAS numbers. Historical aside: CAS was our input contractor for about 6 or 8 years about 1975-1983 or something, so they automatically assigned a CAS number for everything that came in. And so there were CAS numbers: you figured you might be able to search on that and so that's where this group came from. Where we got the coordinates-it was actually the SANSS connection tables, they were converted to a SD format and the programme CORINA [1] from Johann Gasteiger was used to generate 3D coordinates, so that was the first stage of the release.

**Figure 12:**
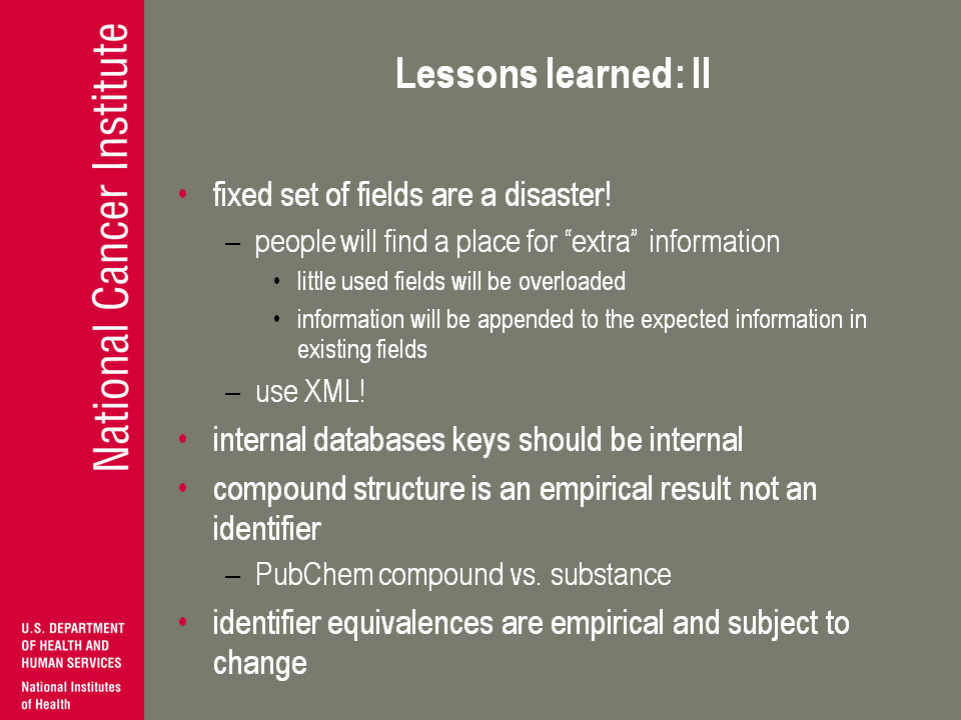
**Lessons Learned: II**.

We had our major conversion, a program called Kekule [2] to look at graphics and try and to chemical structure. The internal system was finished in 2000 and so now when we pull from our company database we are actually pulling MDL format files; so at least it's a format that tries to recognise chemicals structure. Right now we have releases about once a year; we're hoping that in the next year we'll go to a little bit more often than that. The latest release was a few weeks ago and 265 almost 266 thousand structures. The other thing I'll make a point about is, we've been releasing these sets for a long, long time. A lot of people have pulled them up and we have PubChem [3] now. A lot of people, their deposition in the PubChem was basically from a file that they pulled from us that's not documented, and that's fine, it's legal but there are certainly inconsistencies and differences in all kind of things in this data. If you go to PubChem and say 'what's the structure of "something-or-other-amycin"?', and you could look it up and maybe you find ten versions in PubChem. Ten depositions for a compound with that name and maybe you say seven of them have the same actual chemical structure but there's these others that are different. Well I can believe that if seven people think it's this and only two people think it's that, it's probably the seven people that are correct. But it might be that those seven versions have a mistake in them that are propagated because all of them go back to downloading our structures. So it's just a heads up that without the background, without the metadata about where these structures came from, you can potentially get into problems or you can potentially be misled.

(Figure 12) So lessons learned number two. It's one of my pet peeves-a fixed set of fields are a disaster. People are gonna find a place for the information that they need to store no matter what, and if you fixed your set of fields to some gigantic number ('I'm gonna think of everything possibly people are gonna store'), there's always gonna be something you forgot and there's always gonna be a huge number of fields in that case that are never used. So what's the problem? Just stick them in as a field that's never used...but now all your careful documentation of what that field is for is polluted because people don't use it like that! Information will be appended to the expected information in existing fields, so again you have your case when you are plopping some kind of composition data, some kind of stereochemical data into a label on a dummy atom in a structure picture. If that's the place where you can put it, people put it there and you're not gonna stop them. Use XML!

So we go back to, where did I first meet Peter Murray-Rust and why am I so high on XML? It's all because of Peter. I think the first time I interacted with Peter was in 1995, maybe one of the earlier attempts at an internet chemistry poster session, having a chemistry meeting over the internet and I put in a presentation about 3D database searching. And Peter started asking questions basically along the lines of 'can we get to the point where we don't have to do the experiments?' Well gee, if you don't have all the stereochemical information, this and that, and I said 'well I don't think that's a real goal' and I'm thinking to myself 'good God man be reasonable'. Of course in the last fifteen years, that's simply not a thing you say to Peter! I mean he's never gonna be reasonable although he does it in a way that always pushes us. Thinking about this-I'm not sure 100% comes through in this talk-that a lot of this stuff really is Peter's influence, making sure we are driven in directions that are gonna be useful.

Internal database keys should be internal. When you start to make your internal keys meaningful in the external world you lose your flexibility and maintaining really good internal consistency-you should make that primary. The compound structure is an empirical result; it is not an identifier and again I don't know whether PubChem got the terminology right but their distinction between a compound and the substance I think is extraordinarily important when you talk about chemical structure data and bioassay data-I'll give an example of that

**Figure 13:**
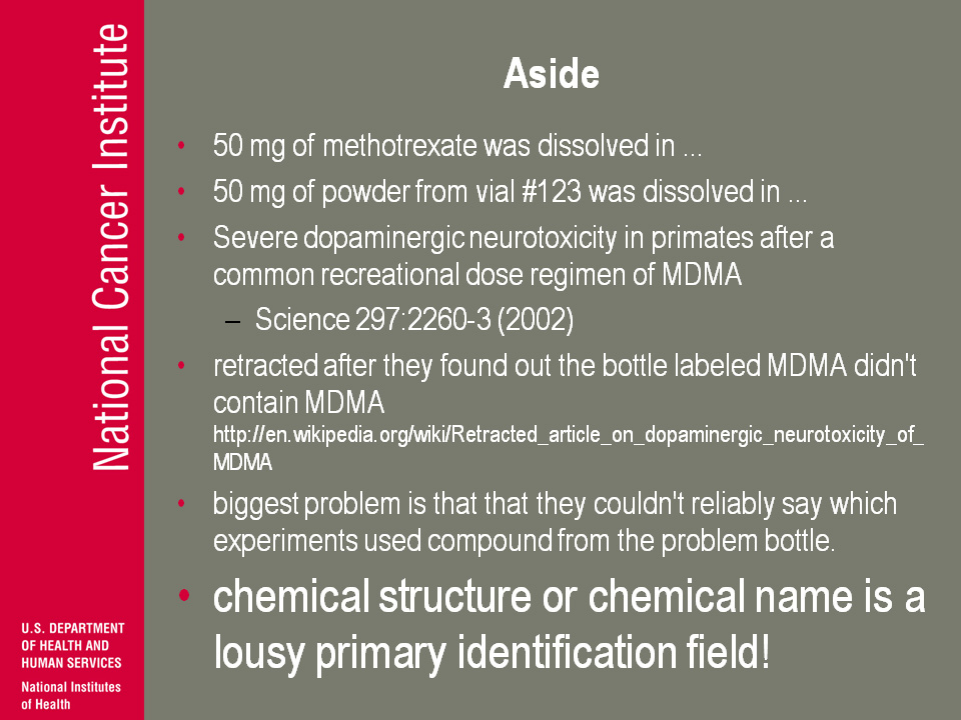
**Aside**.

The other thing I've learned is identifier equivalencies. So you have a CAS number, you have a NSC number, you have this, you have a name, you have all kinds of stuff. Identifier equivalencies are pivotal too: there are claims people made-NSC27 is the same as CAS number blah blah blah. We can use those labels interchangeably-that's a claim, and again various people make various claims and sometimes the claim is wrong and sometimes the claim is misleading. So if you don't understand and can't manage where those claims come from and have access to them you're gonna eventually run into problems. I have an aside:

(Figure 13) Think about the difference of writing in a laboratory notebook '50 milligrams of methotrexate' or '50 milligrams of a powder from vial number 123'. To give a concrete example, there's a paper published in Science about MDMA (3,4-Methylenedioxymethamphetamine, ecstasy). They retracted the paper after they found out the bottle they had used that was labelled with MDMA did not actually contain MDMA, but methamphetamine.

**Figure 14:**
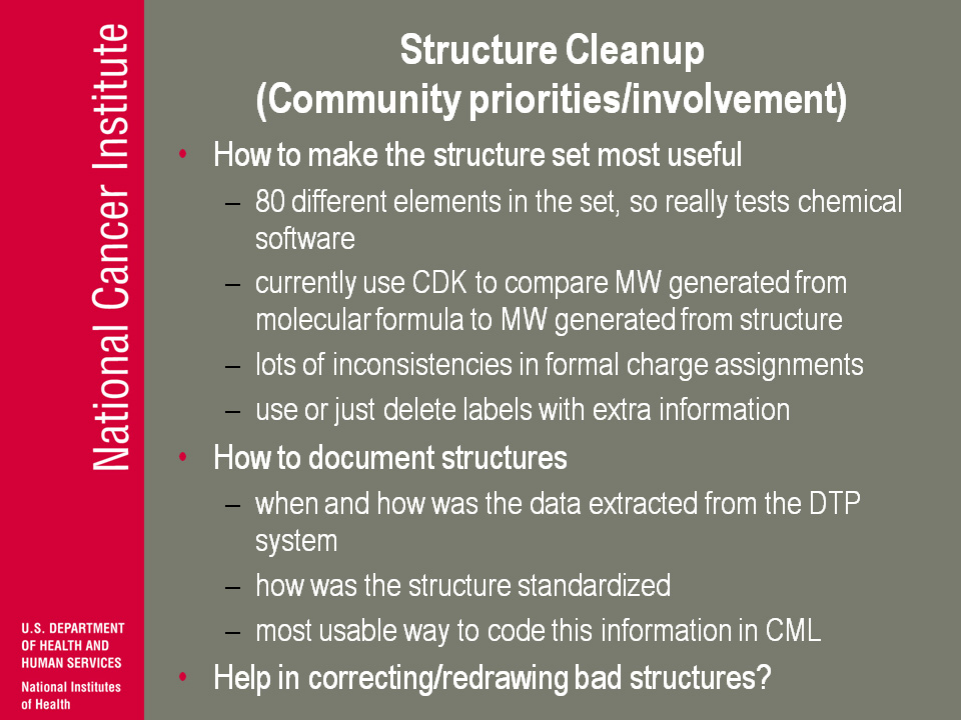
**Structure Cleanup (Community priorities/involvement)**.

The biggest problem when you make mistakes: it's embarrassing, you should double check, but the biggest problem here was is they couldn't even reliably say which experiments were affected by this bottle because they had done this in their notebooks. They had not identified the bottle, they just said 'yeah we used...' so chemical structure, chemical name is just a lousy primary identification field, and you're really gonna run the risk of corrupting data and not having full control of data if you don't understand this difference.

(Figure 14) A couple of these things in the last few slides are labelled community priorities and/or involvement. What I mean by that is these are things I've been thinking of that I think are useful or can potentially be useful, and obviously in this day and age if you can get people to help you actually do it that's fantastic, jump in, let's go. But for our purposes even expressing a notion 'is this a high priority or low priority', 'what kind of thing is useful to us', that helps us with our limited resources say 'well gee a whole lot of people wanna do this so maybe that is where we put our cut off' so when I say this I really would like feedback on any level.

One of the things I have been worried about is how to make the structure set, the data structure, the chemical structure set, more useful. There are at least eighty different elements in the set so it really does exercise any kind of chemical software. It's not just carbon, nitrogen, oxygen, blah blah blah-there's I think 300 tin compounds. We currently use the Chemical Development Kit [4] to compare the molecular weight generated from the molecular formula to the molecular weight generated from the structure. You go back to this problem with SANSS: did it try and represent the complete molecule or not, only some little bit? The molecular formula in our data base was always entered independent of the structure, and so if these two things match you have a little bit of added confidence that the structure that came out really does intend to represent the full structure. If they don't match then well maybe you have a problem. A lot of times when they don't match, it comes down to this: inconsistencies in formal charge assignments. A lot of times it is easy to see how you would clean that up: the molecular formula says 'dot-CL', the structure says 'CL-minus': OK, I understand that. Some of them are not so clear. Do you try and use what I mentioned before-do you try and use the information from these dummy atom labels or do you just forget about them?

**Figure 15:**
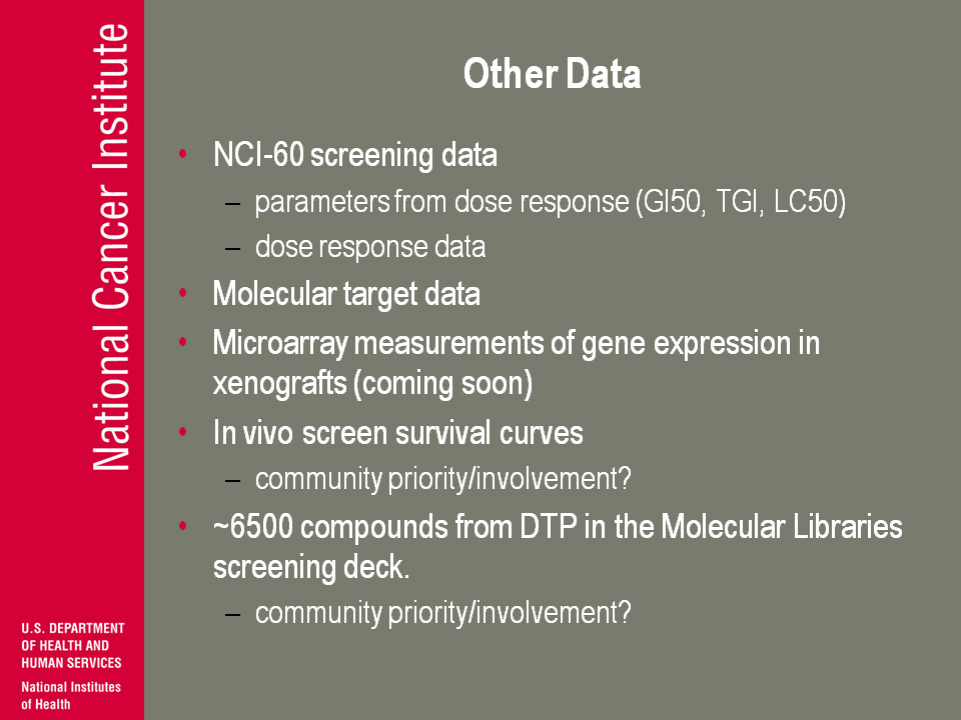
**Other Data**.

How to document the structures-when and how was the data extracted, did it come from us, what kind of algorithms were used to do any kind of clean up or any kind of comparison. Are there beginning to get ways that people would like to see structures standardised more? The most useful way to code this is in the chemical mark-up language-I'm beginning to think that the best way to just to do it and let things evolve. But if people have strong opinions on how to represent some of this and potentially help and correcting or withdrawing bad structures from the community-we had a student in the summer crank out about 400 compounds in a couple of weeks-it might be something people may be interested in.

(Figure 15) Other data. We have our NCI screening data-this is growth inhibition in human tumour cell lines. We have this both as calculated parameters from a full dose response curve, and as a full dose response curve. I mentioned molecular target data: there's microarray of gene expression in vivo in xenografts, which I think could be very important; that data is not quite public yet but it should be soon. We have in vivo survival screens curves from those old mouse model screens, so if anybody's interested in developing software or display tools for looking at survival curves we probably have something like 200 or 250 thousand survival curves. That's publicly available.

**Figure 16:**
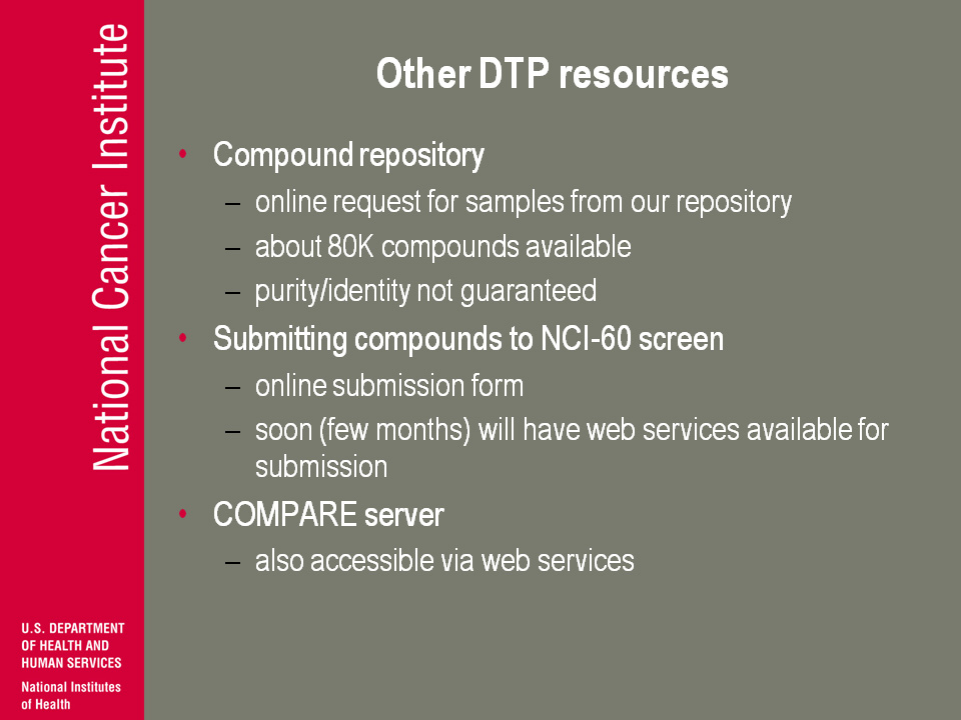
**Other DTP resources**.

**Figure 17:**
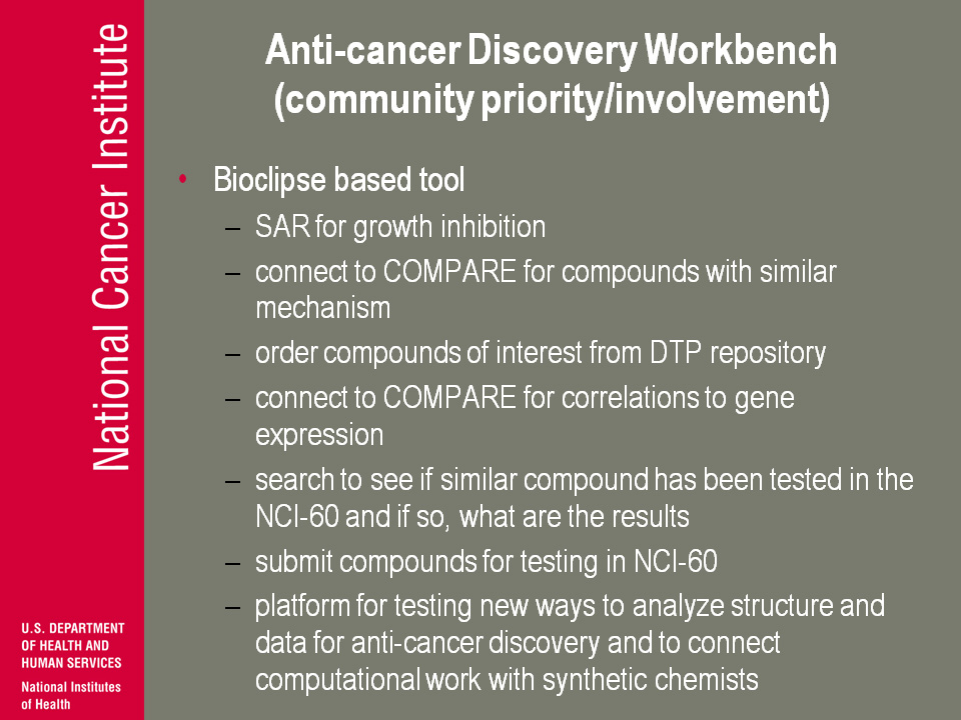
**Anti-cancer Discovery Workbench (community priority/involvement)**.

**Figure 18:**
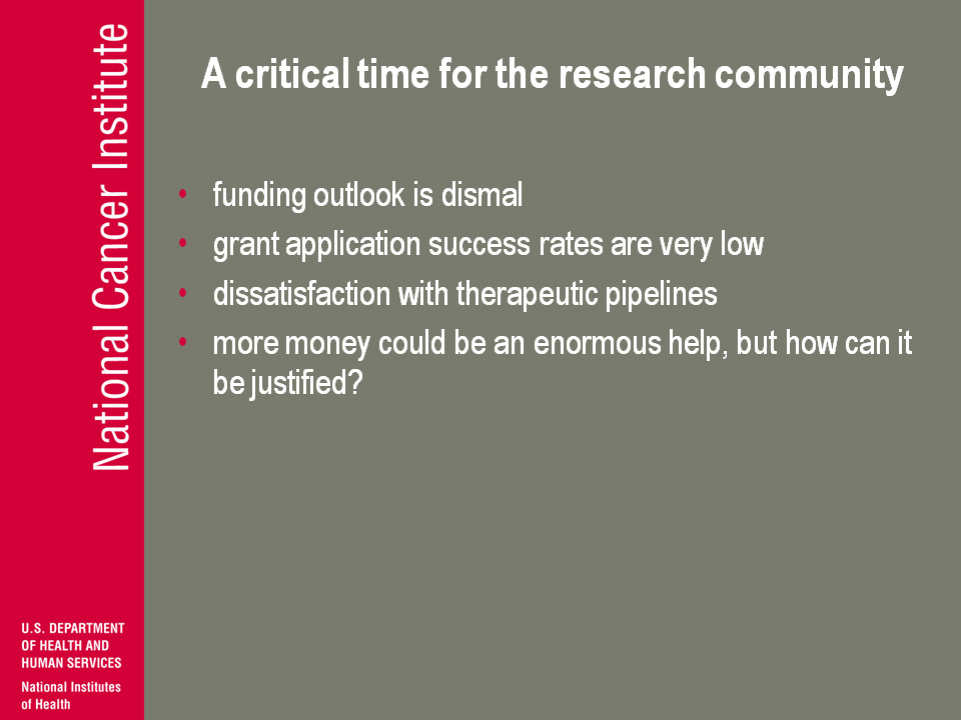
**A critical time for the research community **.

**Figure 19:**
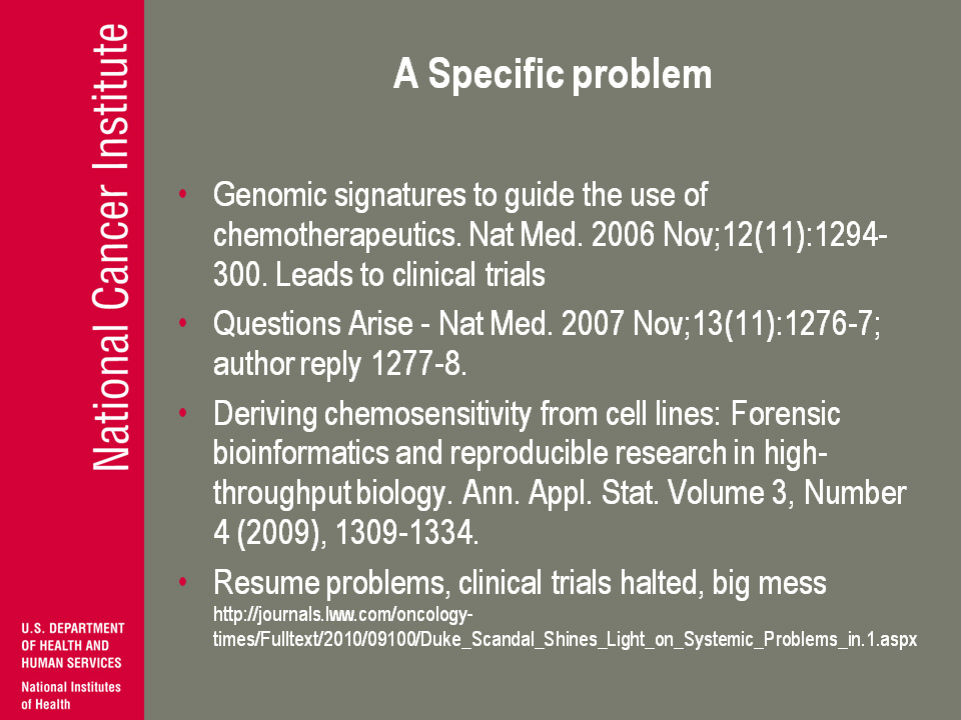
**A Specific problem**.

**Figure 20:**
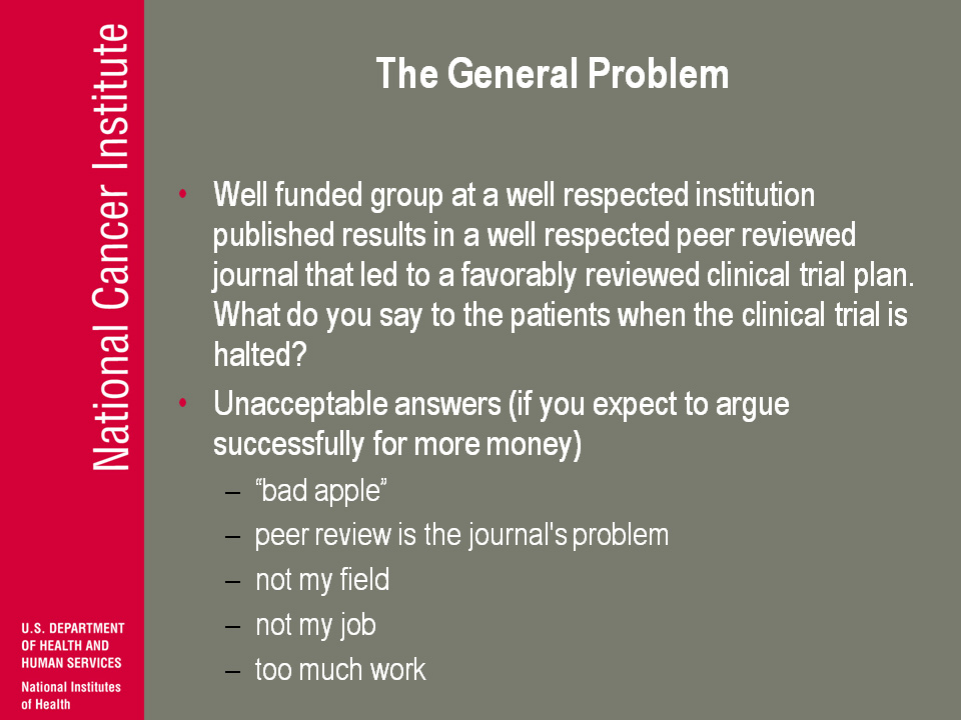
**The General Problem**.

**Figure 21:**
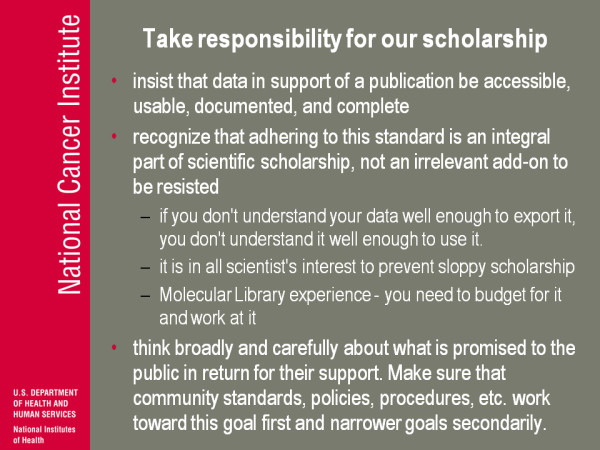
**Take responsibility for our scholarship**.

I probably don't have time to talk about this but we have about 65 hundred compounds from our inventories now in the molecular library screening deck, so we have the ability not only to associate NCI-60 data (let's say a pattern of activity in the NCI-60 cells) but then in some cases we have 2 or 3 hundred assays in the molecular library in PubChem that can be related to them. We haven't really started to develop ways to bring all those things together and try to find ways to best utilise them. Again, they came from us so again we have a guarantee that the NCI-60 data and the molecular library screening data actually all came from the same sample.

(Figure 16) There are other DTP resources. We have a compound repository, as I mentioned before. For a good chunk of our screening, we wanted a couple of grams. If the molecule was abandoned fairly early on we had a gram left, so there are about 80,000 compounds for which I think at least formally we have a gram in our inventory. We have an online sample request for that. We have not for the most part identified to run any analytics on this, although that's changing. You can submit compounds to the screen-we have an online submission form. In a few months we'll have web services and one of the things I'm excited about here is talking to, say, Alex [Wade]and a few people about ways to use these web services where you can manage your submissions and accounts in a Word program or something similar. We also have a COMPARE service server set up so you can do these calculations. We have put them up as web services but we haven't really taken advantage of that, building alternative interfaces to this and alternative ways of putting it together with other things. We haven't really gone beyond that.

(Figure 17) In terms of what my fantasy-well, hopefully it's not a fantasy (but you know...)-how about an anti cancer discovery workbench? Sort of a Bioclipse [5] where you can do structure activity for growth inhibition, connect to COMPARE to look for compounds with similar patterns and mechanism. You could order compounds from DTP, you could connect to COMPARE, you can do correlations to COMPARE, not only look at growth inhibitions versus growth inhibition but growth inhibition versus molecular target data so you can prepare growth inhibition to gene expression, see if a compound has been tested in NCI-60, submit compounds: basically a platform for testing new ways to analyse structure and data, and also to connect people that do computational work with synthetic chemists. If the synthetic chemist is sitting at his bench using this to submit the compounds to NCI the other tools there would be available to them to do ways of hopefully prioritising and maximising their chances of getting good structures submitted.

(Figure 18) And in the last few minutes just talk a little bit about sort of philosophy. So the it's a critical time for the research community-the funding outlook is dismal, everybody knows that grant applications success rates are very low, dissatisfaction with therapeutic pipelines, nobody is happy with the rate and what looks like the therapeutics. You can say 'well, more money could be a big help', and it could save some things in some ways but the question is how you justify it.

(Figure 19) Let me give you a specific problem and this is very dear to my heart because this should be exactly what the developmental therapeutics programme is enabling. There is a paper, published in Nature Medicine in 2006, and it looked for genomic signatures to guide the use of chemotherapeutics. They started with our NCI-60 data and our microarray data, fantastic! They downloaded it and they could do something: they went beyond it, they got a Nature Medicine paper and it led to clinical trials. Other questions arose-a letter to Nature Medicine back and forth, but then a group at MD Anderson [Cancer Center] couldn't quite figure out how they got the results they got. The group at MD Anderson can download our data as well but couldn't quite balance it; the first group would cooperate but they couldn't figure things out. It led finally to this Annals of Applied Statistics paper where the group at MD Anderson laid out (they called it 'forensic bioinformatics'), what they tried to do, how they tried to go about it and the fact that they couldn't really be sure in what was going on. In taking public data, publishing a paper but not being able to connect the dots so you know you can... Here's one particular URL that has a fairly reasonable overview: resume problems arose, clinical trials halted, a very big mess and it's a huge problem.

(Figure 20) What you have is an example of what could be a general problem: it's a well funded group in a well respected institution, published results in a peer review journal, favourably reviewed clinical trial plan. What do you say to the patients when a clinical trial is halted? 'Oops, sorry, my bad, no problem. By the way can you write your congressman and tell them they got to double our NIH budget?' There is a real disconnect there. So what kind of answers can you give? Oh you know "it was a bad apple"; "this guy is bad"; "he lied so he probably faked it"; blah blah blah. I don't think any of these are acceptable, they are primarily ways to avoid responsibility by making it a specific problem, but there's too many interactions with the entire research community here to avoid a more general responsibility. The basic fundamental problem is not any of these (no it's not my problem excuses); the problem is the research community did not demand full accountability, the parameters for all this review was not something that was at all able at all to catch this.

(Figure 21) So I call it, Peter says something sometimes about 'take back our scholarship', I say 'take responsibility for our scholarship': insist the data supporting publications be accessible, useable, documented and complete, and recognise adhering to this standard. Is what science is, it's not an add-on, it's not an extra burden from on high. I claim if you don't understand your data well enough to export it, you don't understand your data well enough to use it, and it's in all scientists' interests to prevent sloppy scholarship. I don't think anybody in the research community benefits from the mess of the genomic signature paper, whether you were directly a part of it or not. I work in the molecular library. There is, and again this is indirectly a tribute to Peter, the ethos and the setup of how data was handled in the molecular library. I strongly argued for [this] and my arguments were influenced by Peter's, but the idea is to make sure that from the beginning the data was released as soon as it was verified. But you need to budget for it and you need to work at it and administratively you need to keep at these people to do it and it's been constant: it works but it doesn't 'just happen'.

**Figure 22:**
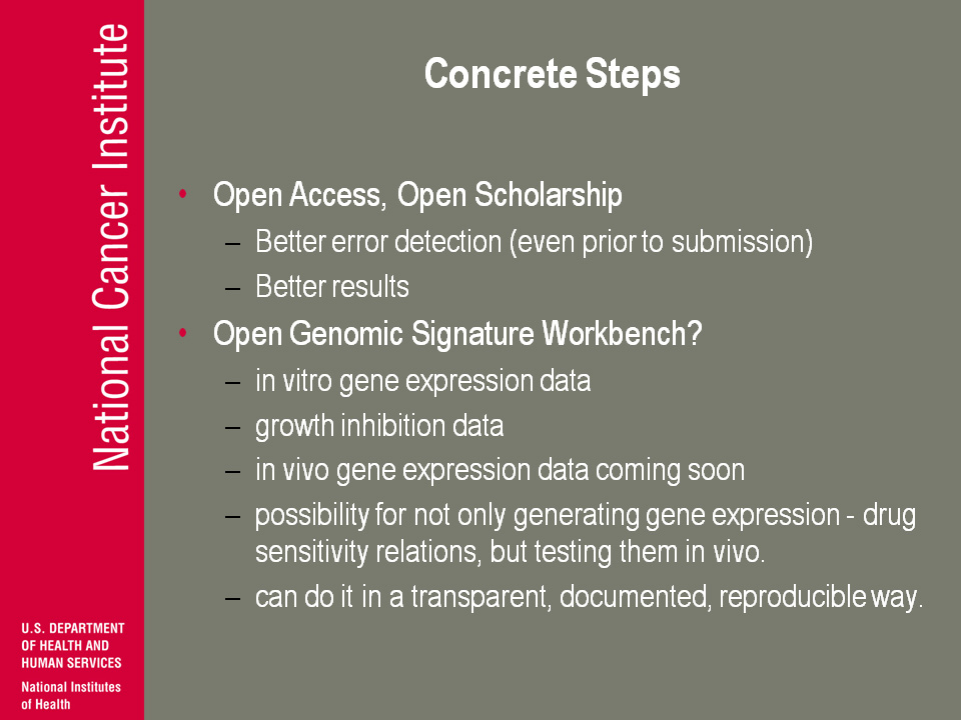
**Concrete Steps**.

So in general you know I think you need to think broadly and carefully about what is promised to the public in return for their support and you have to make sure that all these community standards policies and procedure work toward that goal first the goal of delivering what you are claiming the public benefits from, and all the other goals are secondary: all the prestige, the money and all that stuff.

(Figure 22) Concrete steps: Open Scholarship, Open Access. There's a lot of people here that are gonna talk more than I about that, but I think that from my perspective, in addition to a kind of philosophical approach, it's a very practical approach where you get better error detection, potentially better results, better able to connect.

**Figure 23:**
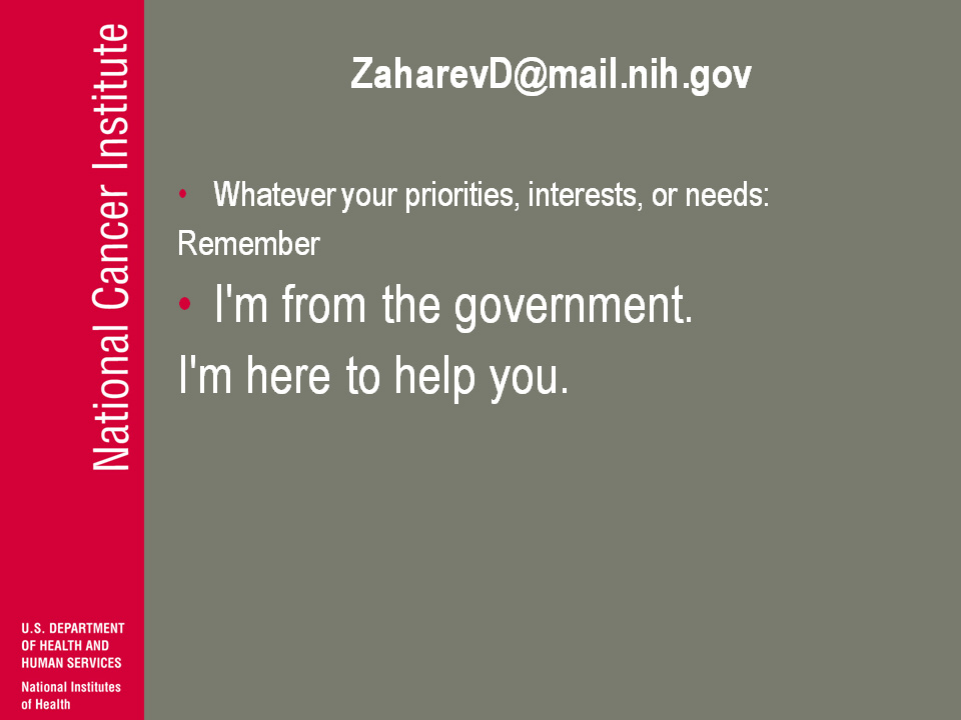
**Conclusion**.

The other thing I'm thinking about is whether can we actually take that [genomic signature] fiasco and turn it around and say 'here's how we would do it with Open data', 'here's how we would do it in a more documented way' and have maybe an Open Genomic Signature Workbench, so we have in vitro gene expression, we have growth inhibition data, we're going to publicly soon have in vivo gene expression data so we have all the pieces. NCI has the xenograft testing possibilities so we have all the pieces for not only generating drug expression, drug sensitivity relations but *testing *them before you start to go to the clinic and you can do it in a transparent, documented and reproducible way. We can show people how it should be done.

(Figure 23) So here's my email address. All those things-remember whenever your priorities, interests, needs: I'm from the government, I'm here to help you.

[Applause]

Question from Egon Willighagen: Are all the characterizations other than the gene expression data of the NCI-60 publicly available?

Dan Zaharevitz: Yes-we have lots of other different characterisations so we have metabolomics data we have enzyme activity measurements but in terms of number of data points the largest set of data in the molecular targets set of data is gene expression data just by number but there's a lot of other things in there as well

## Endnotes

### Endnote 1

Unfortunately the first part of the recording was corrupted, so the talk appears to begin at slide 6, 'At a critical time'.

### Endnote 2

PMR: The NCI research was for many years the outstanding example of Open, publicly financed research and data collection. It stemmed from President Nixon's "war on cancer" which captured the spirit of the moonshots but also shows that biology is tougher than physics.

### Endnote 3

PMR: The systematic testing of public and private compounds was a key strategy for NCI DTP.

### Endnote 4

PMR: Ken Paull's contribution to DTP was dramatic. The COMPARE program is one of those archetypal tools which is both very simple and very powerful. It's a table "browser" for the DTP data with compounds == rows and screens == columns. By tabulating hits compounds can be compared by activity in screens and screen can be compared ***by activity***of compounds. And it emphasizes the importance of having lots of data, carefully aligned, and the tools to manipulate it.

### Endnote 5

PMR: In the early years data was "paper". Chemical structures were hand drawn.

## References

[B1] CORINA-Fast Generation of High-Quality 3D Molecular Models http://www.molecular-networks.com/products/corina

[B2] McDaniel JR Balmuth JR Kekule: OCR-optical chemical (structure) recognition J Chem Inf Comput Sci 1992 32 373 378 10.1021/ci00008a018

[B3] PubChem http://pubchem.ncbi.nlm.nih.gov/

[B4] The Chemistry Development Kit http://sourceforge.net/projects/cdk/

[B5] Bioclipse http://www.bioclipse.net/

